# Population Pharmacokinetic Analysis and Modelling of Serum Uric Acid Dynamics in Patients Treated with Favipiravir

**DOI:** 10.3390/ph19071008

**Published:** 2026-06-29

**Authors:** Tomona Yamada, Hitoshi Kawasuji, Chika Ogami, Chihiro Hasegawa, Makito Kaneda, Daichi Yamaguchi, Satofumi Iida, Takahiko Aoyama, Yoshihiro Yamamoto, Yasuhiro Tsuji

**Affiliations:** 1 Laboratory of Clinical Pharmacometrics, School of Pharmacy, Nihon University, 7-7-1 Narashinodai, Funabashi, Chiba 274-8555, Japan; phto21242@g.nihon-u.ac.jp (T.Y.); ogami-chika@umin.ac.jp (C.O.); chihiro.hasegawa@merck.com (C.H.); daichi.yamaguchi@shionogi.co.jp (D.Y.); aoyama.takahiko@nihon-u.ac.jp (T.A.); 2 Department of Clinical Infectious Diseases, Graduate School of Medicine and Pharmaceutical Science, University of Toyama, 2630 Sugitani, Toyama 930-0194, Japan; kawasuji@med.u-toyama.ac.jp (H.K.); kane2214@med.u-toyama.ac.jp (M.K.); yamamoto@med.u-toyama.ac.jp (Y.Y.); 3 Laboratory of Clinical Pharmacology, Yokohama University of Pharmacy, 601 Matano-cho, Totsuka-ku, Kanagawa 245-0066, Japan; s.iida@yok.hamayaku.ac.jp

**Keywords:** favipiravir, hydroxylated metabolite, population pharmacokinetics, uric acid, pharmacodynamics, hyperuricemia

## Abstract

**Background**: Hyperuricemia is an adverse effect frequently observed during favipiravir treatment. The time course, from uric acid elevation to recovery, and quantitative relationship between drug exposure and changes in serum uric acid levels remain insufficiently characterized. We investigated the pharmacodynamic mechanism of uric acid elevation and described its time course by population pharmacokinetic and pharmacodynamic modelling. **Methods**: Patients who received favipiravir for coronavirus disease 2019 or severe fever with thrombocytopenia syndrome were retrospectively evaluated. The pharmacokinetics of favipiravir were described by a one-compartment model with first-order absorption and elimination. Metabolite concentrations were predicted based on previously reported values. Changes in serum uric acid levels were described by a turnover model with zero-order production and first-order elimination. The drug effect was implemented as inhibition of the uric acid elimination process. Simulations based on the final model were performed for 10 consecutive days after the clinical regimen, with a 21-day follow-up. **Results**: The final model supported the inhibition of uric acid elimination by favipiravir and its metabolite. Regarding simulations, serum uric acid levels reached a median peak of 6.93 mg/dL at 6.7 days after treatment initiation and returned to pre-treatment levels within 4.0 days after treatment discontinuation. **Conclusions**: This combined population pharmacokinetic and pharmacodynamic turnover model quantified favipiravir-associated increases in serum uric acid levels and showed a transient profile with rapid recovery after drug discontinuation. These findings underscore the need for monitoring serum uric acid levels during favipiravir treatment, particularly in patients at a higher risk of gout.

## 1. Introduction

Favipiravir (FPV) is a broad-spectrum antiviral agent that inhibits the RNA-dependent RNA polymerase of RNA viruses [1,2,3]. As the catalytic domain of RNA-dependent RNA polymerase is conserved among multiple RNA viruses, FPV exhibits antiviral activity against a wide range of RNA viruses [1]. In Japan, FPV was approved in 2014 for treating novel or re-emerging influenza virus infections, limited to cases in which other anti-influenza agents are ineffective or insufficient [4,5]. During the coronavirus disease 2019 (COVID-19) pandemic, FPV was administered in clinical and observational studies in Japan and other countries [2]. In 2024, FPV was approved in Japan as an antiviral agent for severe fever with thrombocytopenia syndrome (SFTS) [4].

FPV is well absorbed from the gastrointestinal tract after oral administration [5]. It is primarily metabolized to the favipiravir hydroxide (metabolite 1; M1) by hepatic aldehyde oxidase with partial contribution of xanthine oxidase, followed by renal excretion [5]. FPV alters its pharmacokinetics via aldehyde oxidase auto-inhibition. As a result, exposure may vary depending on dose and duration [5]. Interindividual differences in aldehyde oxidase activity may contribute to the variability in plasma FPV concentrations. Pharmacokinetic studies in patients with COVID-19 reported substantial interindividual variability in FPV exposure [6,7]. In severe cases, the trough concentrations were frequently below the lower limit of quantification. Patients with mild-to-moderate disease exhibited marked decreases in trough concentrations and area under the curve (AUC) between days 2 and 4 after treatment initiation [6,7].

Serum uric acid homeostasis is maintained by a balance between urate production and excretion, and urate is mainly eliminated via the kidneys [8,9,10]. Particularly, multiple transporters in the renal proximal tubule contribute to urate reabsorption and secretion, including reabsorption via urate transporter 1 and transport via organic anion transporters 1 and 3, which are involved in regulation of serum uric acid levels [8,10,11]. Hyperuricemia is frequently observed as a common adverse effect of FPV treatment [4,12]. As a potential mechanism, FPV and M1 have been reported to affect urate transporters such as urate transporter 1 and organic anion transporters 1 and 3, potentially reducing urate excretion [5,12]. In clinical studies including patients with COVID-19, baseline serum uric acid level (UAbase) and steady-state FPV concentrations were associated with uric acid elevation [13,14]. However, the longitudinal time course of uric acid elevation and recovery after FPV administration has not been well characterized, and quantitative pharmacokinetic and pharmacodynamic analysis linking FPV exposure to changes in serum uric acid levels remains limited. The aims of the present study were (i) to perform a population pharmacokinetic and pharmacodynamic analysis of FPV and evaluate the association between exposure and changes in serum uric acid levels and (ii) to characterize the time course of elevation of serum uric acid levels and recovery after FPV administration.

## 2. Results

### 2.1. Patients and Data Sources

A summary of patient characteristics is presented in Table 1. A total of 23 patients, consisting of 12 male and 11 female patients, who received FPV and 92 observed FPV concentrations were included in the final analysis. The median age, total body weight, and administration period were 69 years, 68 kg, and 13 days, respectively.

### 2.2. Population Pharmacokinetics

**Figure 1:**
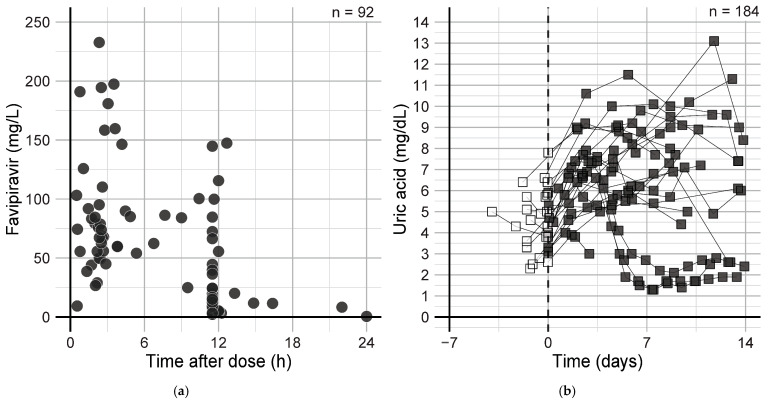
Observed favipiravir concentrations and longitudinal uric acid profiles before and after treatment initiation: (**a**) Scatter plot of observed favipiravir concentrations (mg/L) versus time after dose (h) (*n* = 92). (**b**) Individual patient uric acid measurements (mg/dL) plotted against time (days) (*n* = 184). Day 0 (dashed line) indicates treatment initiation. Open and filled squares represent pre-treatment and post-treatment values, respectively. Repeated measurements from the same patient are connected by lines to depict within-patient trajectories across treatment initiation.

The time course of observed FPV concentrations before and after dosing is shown in Figure 1a. Alanine aminotransferase was evaluated as a covariate on CL_FPV_/F. However, no statistically significant improvement was observed (ΔOFV = 0.631, df = 1, *p* = 0.427). Similarly, age was not statistically significant (ΔOFV = 0.004, df = 1, *p* = 0.950). The final pharmacokinetic model parameters are shown in Equation (1).

(1) $${CL}_{FPV}/F\;(L⁄h)=1.31\times F_{SIZE}\;V_{FPV}\;(L)=10.4\times F_{SIZE}\;{CL}_{M1}\;(L⁄h)=16.3\;(FIXED)\times RF\times F_{SIZE}\;V_{M1}\;(L)=6.44\;(FIXED)\times F_{SIZE}\;RF=1+0.0095\;(FIXED)\times (CLcr-CLcrSTD)\;ka=\frac{ln2}{1.15}$$

Goodness-of-fit plots based on the final pharmacokinetic model are shown in Supplementary Figure S1. Predicted FPV concentrations were consistent with observed concentrations (Figure S1a,b), and the plots were distributed close to the trend line at y = x. R^2^ values for these plots were 0.505 (a) and 0.788 (b). Most conditional weighted residuals fall within ±3 and were evenly distributed around y = 0 (Figure S1c,d) [15]. η-shrinkage for the final model was 5.37% for CL_FPV_/F.

**Figure 2:**
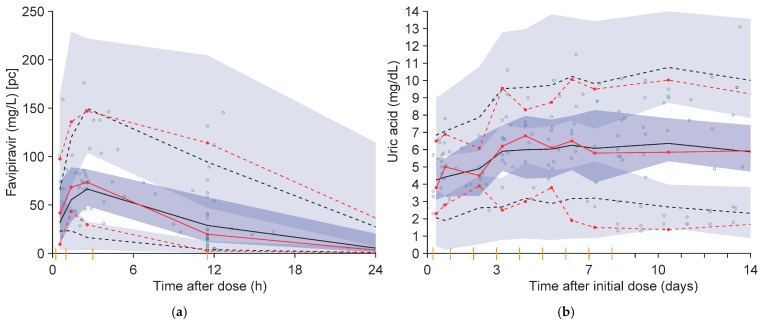
Visual predictive check (VPC) based on the final population parameter estimates: (**a**) Prediction-corrected VPC (pcVPC) for favipiravir concentrations. (**b**) Standard VPC for uric acid. Circles represent observations. Solid lines show the observed (red) and simulated (black) medians. Dashed lines show the 5th and 95th percentiles (central 90% interval) of the observations (red) and simulations (black). Shaded areas indicate the 95% confidence intervals of the simulated percentiles. The yellow lines on the x-axis show the mid-point of data bins used in the construction of the VPC.

The bootstrap results are shown in Table 2. All final pharmacokinetic parameter estimates were similar to the average of the bootstrap distributions and fell within the empirical bootstrap 95th percentiles. No marked imprecision was found in the RSE%. Model evaluation by pcVPC also confirmed acceptable agreement between the observed data and the model-based simulated values (Figure 2a). The median of the observed values was within the 95% CI of the predicted values.

### 2.3. Population Pharmacokinetic and Pharmacodynamic Modelling

The time course of serum uric acid levels is shown in Figure 1b. A total of 184 serum uric acid levels were available. UAbase was available for 18 of 23 patients, with a median value of 4.43 mg/dL. Only one patient had a pre-treatment serum uric acid level above the reported urate crystallization threshold (6.8 mg/dL) [16,17].

The Imax model was selected to describe inhibition of the uric acid elimination process. The linear model did not provide a significant improvement in model fit. The coefficient α was estimated to assess a potential synergistic contribution and was greater than zero. The model was then refitted with α fixed to 0 to assume additivity. The synergistic model did not provide a statistically significant improvement over the additive model (ΔOFV = 2.78, df = 1, *p* = 0.095), and the additive model was therefore retained.

The bootstrap results are shown in Table 2. The final pharmacodynamic parameter estimates were consistent with the bootstrap mean estimates and fell within the empirical 95% bootstrap percentile range. No marked imprecision was found in the %RSE. The standard VPC showed acceptable agreement between the observed data and model-based simulations (Figure 2b). The median of the observed values fell within the 95% CI of the predictions.

### 2.4. Simulation

**Figure 3:**
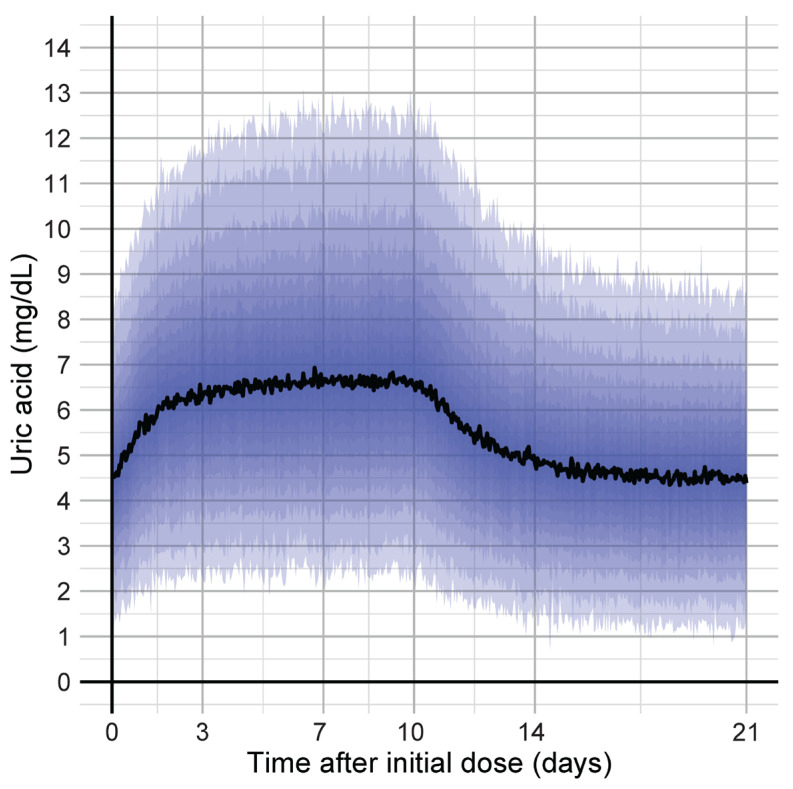
Simulated time course of uric acid after standard-dose favipiravir based on the final model. The solid black line represents the median prediction. Purple shaded bands represent prediction percentiles in 10% increments (from the 10th to the 90th percentile), with darker shading indicating more central percentiles.

The simulated time course of serum uric acid levels following FPV administration based on the final model is shown in Figure 3. The serum uric acid level reached its maximum at 6.7 days after treatment initiation, with a median peak value of 6.93 mg/dL. The time required for the serum uric acid level to return to UAbase after treatment discontinuation was 4.0 days.

## 3. Discussion

In this study, a population pharmacokinetic and pharmacodynamic analysis was performed in patients treated with FPV to quantitatively evaluate the relationship between FPV exposure and changes in serum uric acid levels. The increase in serum uric acid levels associated with FPV administration was adequately described as inhibition of the uric acid elimination process. Consistent with previous reports, the model supported the contribution of M1 exposure to elevation of serum uric acid levels. Regarding the simulation analysis, the serum uric acid level reached a maximum at approximately 7 days after treatment initiation and returned to UAbase approximately 4 days after treatment discontinuation. This study characterized the longitudinal time course of changes in serum uric acid levels during and after FPV treatment and provides information relevant to monitoring and management of serum uric acid levels in clinical practice.

Regarding the analysis population, at the time of the ethics committee review during the early phase of the COVID-19 pandemic, knowledge and evidence on background factors to be considered for exclusion criteria were limited. Therefore, an inclusive approach was adopted to incorporate clinical data as comprehensively as possible, and all cases in the analysis population were included.

FPV pharmacokinetics have been reported using noncompartmental analysis and one-compartment models [5,18,19,20,21,22,23]. In this study, the pharmacokinetics of FPV were described by a one-compartment model. This structural model is consistent with previously reported population pharmacokinetic analyses and is considered to reflect the basic disposition characteristics of FPV [22,23]. The number of patients (*n* = 23) and FPV concentration samples (*n* = 92) were limited, which may constrain the power to detect covariate effects and the precision of BSV estimates. Accordingly, a parsimonious pharmacokinetic model was constructed, avoiding overestimation of random effects and excessive inclusion of covariates. No major discrepancies were observed in the goodness-of-fit plots, bootstrap analysis, or VPC and pcVPC, supporting the robustness and adequacy of the final model within this dataset.

Considering FPV, previously reported values for CL_FPV_/F and Vd_FPV_ range from 0.47 to 19.6 L/h and from 4.85 to 41.6 L, respectively [5,18,19,20,21,22,23]. In this study, the estimated CL_FPV_/F and Vd_FPV_ were 1.31 L/h and 10.4 L, respectively, which were within the ranges reported in previous pharmacokinetic analyses [22,23]. The elimination half-life calculated based on CL_FPV_/F and Vd_FPV_ was 5.46 h, which was comparable to that reported in previous population pharmacokinetic analyses [22,23]. Previous studies in COVID-19 populations indicate that severe inflammation and physiological changes related to the disease may influence drug exposure [22,24]. In this cohort, C-reactive protein levels showed mild elevations, whereas white blood cell counts were generally within the normal range, and albumin levels did not show substantial decreases (Table 1) [25,26]. Therefore, within the clinical range represented in this study, the influence of disease status on favipiravir pharmacokinetics was unlikely to be substantial, and we did not incorporate covariates related to disease status into the model.

In this study, a turnover model was used to describe uric acid kinetics, with zero-order production and first-order elimination [27]. The FPV-associated increase in serum uric acid levels was modelled as inhibition of the uric acid elimination process. This modelling approach has also been used in population pharmacokinetic and pharmacodynamic analyses of other drugs affecting uric acid and was considered appropriate for describing physiological uric acid homeostasis in the present analysis [28,29].

The proposed mechanisms for serum uric acid elevation include inhibition of tubular urate secretion and enhanced urate reabsorption in the proximal tubule. However, the underlying mechanisms remain unclear [12]. As separately quantifying the contributions of secretion inhibition and enhanced reabsorption from the present data was difficult, the effect was modelled parsimoniously as inhibition of the overall uric acid elimination process. The inhibitory effects of FPV and M1 were not supported as synergistic in this analysis, and the model assuming additive effects provided an adequate fit.

As serum uric acid levels ≤7 mg/dL are generally considered below the upper limit of normal, the UAbase estimate (4.43 mg/dL) was reasonable for evaluating FPV-associated increases in serum uric acid levels [30]. The T1/2_UA_ estimate was 17.6 h, which was comparable to the T1/2_UA_ values reported in previous population pharmacokinetic and pharmacodynamic analyses of other drugs (15.9–21.2 h) [28,29].

In the simulation based on the final model (Figure 3), the serum uric acid level was simulated to increase early after treatment initiation and reached a maximum at 6.7 days (6.93 mg/dL). Although the diagnostic threshold for hyperuricemia is 7 mg/dL, the solubility of monosodium urate is reported to be approximately 6.8 mg/dL, above which the risk of monosodium urate crystal formation and precipitation increases [16,17]. In patients with a history of hyperuricemia, even a modest increase in serum uric acid level may increase the risk of crystal formation. A case of an acute gout flare triggered by FPV has been reported in a patient with a history of hyperuricemia whose serum uric acid level had been well controlled with urate-lowering therapy [31]. Therefore, in patients with a history of hyperuricemia, careful attention should be paid not only to changes in serum uric acid levels during treatment but also to clinical symptoms. In contrast, the serum uric acid level was predicted to return to UAbase within 4.0 days after treatment discontinuation, suggesting that FPV-associated uric acid elevation may be transient. This finding is consistent with previous reports showing a rapid recovery of serum uric acid levels after FPV discontinuation [13,14].

A limitation of this study is that serum M1 concentrations were not measured. As the analysis used model-predicted M1 concentrations based on a previously reported population pharmacokinetic model, the individual quantitative contributions of FPV and M1 to the increase in serum uric acid levels could not be strictly separated. In addition, the effects of BSV in M1 exposure and potential interactions between FPV and M1 on uric acid changes could not be directly evaluated.

## 4. Materials and Methods

### 4.1. Ethics

This study was approved by the Institutional Review Boards of the University of Toyama Hospital (approval numbers: R2020146 and R2012133) and Nihon University School of Pharmacy (approval number: 20-013). This study was conducted in accordance with the Declaration of Helsinki, and patient confidentiality was maintained. Serum FPV concentrations were determined using residual serum samples collected during routine clinical care, which were no longer required for clinical testing.

### 4.2. Patients and Data Sources

This was a retrospective observational study. Patients who received FPV (Avigan^®^, FUJIFILM Toyama Chemical, Tokyo, Japan) for treatment of COVID-19 or SFTS at the University of Toyama Hospital between April 2020 and July 2025 were included. No exclusion criteria were defined. Patient characteristics, including age, sex, body weight, renal function, concomitant medications, dosing times, blood sampling times, and treatment duration were collected from medical records. The dosing regimen was 1800 mg twice daily on day 1, followed by 800 mg twice daily from day 2 onward. The treatment duration was determined by the attending physician according to the patients’ clinical conditions.

### 4.3. Determination of FPV Concentrations and Uric Acid Levels

Serum samples for measurement of FPV concentrations were stored at −80 °C until analysis. Serum FPV concentrations were determined using an absolute calibration method by high-performance liquid chromatography based on a previous report [32]. A reference standard of FPV (CAS No. 259793-96-9) was purchased from Med Chem Express (MCE; Med Chem Express LLC, Monmouth Junction, NJ, USA). Chromatographic separation was performed using an aminopropyl column (Unison UK-Amino, 150 × 3 mm, 3 µm; Imtakt, Kyoto, Japan). The mobile phase consisted of 0.1% acetic acid in water. The flow rate was 0.4 mL/min, the column temperature was 60 °C, and ultraviolet detection was performed at 360 nm. The linearity of the calibration curve was confirmed over the range of 0.1–200 mg/L (R^2^ = 0.999). The lower limit of quantification (LLOQ) and limit of detection were both 0.1 mg/L (coefficient of variation < 10%). Samples below the LLOQ were excluded from the analysis [33].

Serum uric acid levels were measured using the uricase–peroxidase method (Quick Auto Neo UA II; Shino-Test, Kanagawa, Japan). The measurable range was 0.2–200 mg/dL (coefficient of variation < 10%).

### 4.4. Population Pharmacokinetics and Pharmacodynamics of FPV

Population pharmacokinetics and pharmacodynamics analysis was performed using the population pharmacokinetic parameters and data (PPP and D) method [34,35], using the first-order conditional estimation method with interaction (FOCE-I) in the nonlinear mixed-effect modelling software NONMEM^®^ version 7.6.0 (ICON Development Solutions, Gaithersburg, MD, USA). Model execution, bootstrap analyses, visual predictive checks (VPCs), simulations, and result management were performed using Wings for NONMEM. Statistical analyses and graphical processing were performed using R version 4.3.2 (R Foundation for Statistical Computing, Vienna, Austria).

### 4.5. Population Pharmacokinetics

The population pharmacokinetic model for FPV was evaluated using a one-compartment model with first-order absorption and elimination (ADVAN 13, TOL = 9). Based on a previously published model, FPV was assumed to be completely converted to M1 [23]. The estimated pharmacokinetic parameters were clearance of FPV (CL_FPV_/F), volume of distribution of FPV (Vd_FPV_), and absorption half-life (T_abs_). The absorption rate constant (ka) was calculated by dividing the natural logarithm of 2 by T_abs_. The absolute bioavailability (F) was fixed to 1 [5,36]. As serum M1 concentrations were not measured in this study, clearance of M1 (CL_M1_) and volume of distribution of M1 (Vd_M1_) were fixed to previously reported values [23]. Between-subject variability (BSV) was estimated for CL_FPV_/F, whereas Vd_FPV_ and T_abs_ were described using typical values only. BSV was modelled with a log-normal distribution (Equation (2)).

(2) $$Pi=Ppop\times e^{\eta i}$$

Pi is the individual parameter value, Ppop is the population typical value, and ηi is a random variable with mean 0 and variance ω^2^.

The residual unidentified variability (RUV) was modelled with combined proportional and additive errors (Equation (3)).

(3) $$Yij=Ypredij\times \left(1+{RUV}_{PROP}\right)+{RUV}_{ADD}$$

Yij is the jth observed concentration for the ith patient, and Ypredij is the corresponding model-predicted concentration. RUV_PROP_ and RUV_ADD_ are the combined proportional and additive error model components, respectively, with mean 0 and variances σ^2^. For M1, previously reported values were used [23].

### 4.6. Covariate Model

The factor for size (F_SIZE_) was applied to standardize the pharmacokinetic parameters, with an assumption of standard total body weight (TBW) of 70 kg (Equation (4)) [37,38].

(4) $$F_{SIZE}={\left(\frac{TBW}{70}\right)}^{PWR}$$

The allometric exponent (PWR) of F_SIZE_ was fixed to 0.75 for CL_FPV_/F and CL_M1_, and 1 for Vd_FPV_ and Vd_M1_.

Creatinine clearance (CLcr) was calculated using the Cockcroft–Gault formula standardized to a TBW of 70 kg (Equation (5)).

(5) $$CLcr=\frac{\left(140-AGE\right)\times TBW}{72\times Serum\;creatinine\;(\mathrm{mg}/\mathrm{dL})}\left(\times 0.85\;if\;female\right)$$

For FPV, alanine aminotransferase and age were evaluated as covariates on CL_FPV_/F. These covariates were modelled with a power function and standardized to the median values in this study population. For M1, renal function (RF) was included as a covariate on CL_M1_ as previously reported (Equations (6) and (7)) [23].

(6) $${CL}_{M1}={POPCL}_{M1}\times F_{SIZE}\times RF$$

(7) RF=1+TRF×(CLcr−CLcrSTD)

RF was defined as a function of the deviation from the standard CLcr (CLcrSTD, 100 mL/min/70 kg) and the RF effect coefficient (TRF). This coefficient was fixed to the previously reported value [23]. Candidate covariates were limited to patient characteristics and standard laboratory test results that could be systematically extracted from medical records, including renal function measures.

### 4.7. Population Pharmacokinetic and Pharmacodynamic Modelling

The time course of changes in serum uric acid levels following FPV administration was described with a turnover model with zero-order production and first-order elimination (Supplementary Figure S2). The estimated pharmacodynamic parameters were UAbase and the uric acid elimination half-life (T1/2_UA_). The uric acid elimination rate constant (kout) was calculated by dividing the natural logarithm of 2 by T1/2_UA_.FPV-induced elevation of serum uric acid levels was modelled as inhibition of the uric acid elimination process (PDI) (Equation (8)).

(8) $$\frac{dUA}{dt}=Rin-kout\times PDI\;Rin=UAbase\times kout\;PDI=1-Edrug;\;Inhibition$$

The pharmacodynamic model for FPV (Edrug) was either a linear or the maximum extent of drug inhibition (Imax) model (Equation (9)).

(9) $$\begin{array}{c} \mathrm{Linear model}\\ \;\;\;\;\;Edrug={SLOPE}_{FPV}\times C_{FPV}+{SLOPE}_{M1}\times C_{M1}+\alpha \times C_{FPV}\times C_{M1}\\ \mathrm{Imax model}\\ \;\;\;\;Edrug=\frac{Imax\times \left(\frac{{C_{FPV}}^{\gamma_{FPV}}}{{{C50}_{FPV}}^{\gamma_{FPV}}}+\frac{{C_{M1}}^{\gamma_{M1}}}{{{C50}_{M1}}^{\gamma_{M1}}}+\alpha \times \frac{{C_{FPV}}^{\gamma_{FPV}}}{{{C50}_{FPV}}^{\gamma_{FPV}}}\times \frac{{C_{M1}}^{\gamma_{M1}}}{{C50}_{M1}}\right)}{1+\frac{{C_{FPV}}^{\gamma_{FPV}}}{{{C50}_{FPV}}^{\gamma_{FPV}}}+\frac{{C_{M1}}^{\gamma_{M1}}}{{{C50}_{M1}}^{\gamma_{M1}}}+\alpha \times \frac{{C_{FPV}}^{\gamma_{FPV}}}{{{C50}_{FPV}}^{\gamma_{FPV}}}\times \frac{{C_{M1}}^{\gamma_{M1}}}{{{C50}_{M1}}^{\gamma_{M1}}}} \end{array}$$

FPV and M1 were assumed to share the same Imax, and a coefficient α was estimated to evaluate additive versus synergistic contributions of the inhibitory effects. α = 0 was defined as additivity, whereas α > 0 was defined as synergy [39]. Imax was fixed to 1. The protein unbound concentrations of FPV and M1 producing 50% of the maximum inhibition effect (C50_FPV_ and C50_M1_) were fixed to values based on prior in vitro experiments [5,12]. As these values were reported as unbound concentrations, they were converted to total concentrations by dividing by the unbound fraction and were incorporated into the model (Equation (10)).

(10) $${C50}_{FPV}=\frac{{POPC50}_{FPV}}{0.5}\;{C50}_{M1}=\frac{{POPC50}_{M1}}{0.3}$$

UAbase was estimated as an individual parameter in the pharmacodynamic model, and no additional covariate testing was performed.

### 4.8. Model Evaluation and Validation

To test the significance of various factors that influenced the pharmacokinetic and pharmacodynamic parameters, the objective function value (OFV) determined in the NONMEM^®^ fitting routine was used. The difference in OFV (ΔOFV) obtained by comparing each model was asymptotically distributed according to the chi-squared test, with the number of degrees of freedom (df) being equal to the difference in the number of parameters between the two models. The significance level was set at *p* < 0.05 (ΔOFV = 3.84).

Goodness-of-fit plots were used for pharmacokinetic model evaluation. Population mean values were assessed using plots of observations versus population predictions (PRED), and BSV for each pharmacokinetic parameter was evaluated using plots of observations versus individual predictions (IPRED) based on Bayesian estimation. Model fit was further assessed using plots of conditional weighted residuals (CWRES) versus PRED and CWRES versus time after the previous dose. η-shrinkage was evaluated for the final model to assess the reliability of empirical Bayes estimate-based diagnostics [40].

A nonparametric bootstrap was used to estimate uncertainty [41]. The final model was fit repeatedly to 500 additional bootstrap datasets. The average, standard deviation, relative standard error (%RSE) and 95% confidence intervals (CIs) were calculated from the empirical bootstrap distribution and compared with the estimates from the original dataset. A VPC was used to check the distribution of observed and predicted percentiles. The VPC was evaluated by comparing the observed concentrations with 90% percentile intervals (PIs) and 95% CIs simulated from the final parameters. Prediction-corrected VPC (pcVPC) was performed for serum FPV concentrations, and standard VPC was performed for serum uric acid levels.

### 4.9. Simulation

Based on the final model, serum uric acid levels were simulated for 21 days from FPV initiation. The scenario assumed 10 consecutive days of dosing, corresponding to the maximum approved treatment duration for SFTS. The clinical regimen was 1800 mg twice daily on day 1, followed by 800 mg twice daily from day 2 onward. Simulations were performed using the $SIMULATION option in NONMEM^®^ with 500 replicates. The time to peak serum uric acid level and time required for the serum uric acid level to return to baseline after treatment discontinuation were evaluated. Return to baseline was defined as the first time point at which the simulated serum uric acid level fell within ±5% of UAbase.

## 5. Conclusions

This study quantitatively evaluated FPV-associated increases in serum uric acid levels, including the time course from treatment initiation through recovery after treatment discontinuation, using a population pharmacokinetic and pharmacodynamic analysis incorporating the pharmacokinetics of FPV and M1. The analysis reaffirmed that FPV treatment increases serum uric acid levels. However, the magnitude of the increase was not excessive from a clinical perspective, and serum uric acid levels were predicted to recover rapidly after treatment discontinuation. Nevertheless, in patients with a history of hyperuricemia who are at higher risk of gout, serum uric acid levels should be monitored during FPV treatment.

## Figures and Tables

**Table 1:** Demographics and clinical data of patients receiving favipiravir.

				Observation Interval	
		Number	Median	Lower 2.5%	Upper 97.5%
Patients					
^a^	Total patients	23			
^a^	Female	11			
^a^	Male	12			
^a^	Age (year)	23	69	52	88
^a^	Total body weight (kg)	23	68.0	40.2	87.9
^a^	Aspartate aminotransferase (IU/L)	23	46	20	129
^a^	Alanine aminotransferase (IU/L)	23	32	12	80
^a^	Blood urea nitrogen (mg/dL)	23	18.9	6.2	36.5
^a^	Serum creatinine (mg/dL)	23	0.9	0.5	1.5
^a^	Albumin (g/dL)	23	3.7	2.4	4.5
^a^	White blood cell count (/μL)	23	4830	1185	9602
^a^	C-reactive protein (mg/dL)	23	3.12	0.30	14.59
	Administration period (days)	23	13	2	14
Favipiravir total concentrations					
	Observed concentration (mg/L)	92	47.2	0.2	193.5
	BLQ (<0.1 mg/L)	12			
Uric acid (mg/dL)		184	5.8	1.7	10.1

^a^ Before Favipiravir administration. BLQ, below the lower limit of quantification.

**Table 2:** Comparison of population pharmacokinetic and pharmacodynamic parameter estimates for the final model with estimates from 500 bootstrap samples.

				Bootstrap Sample Estimates			
					95% CI		
Parameter	Description	Unit	Final Model Estimate	Average	Lower 2.5%	Upper 97.5%	%RSE
Population mean							
*Pharmacokinetics*							
CL_FPV_/F	Clearance of favipiravir	L/h	1.31	1.31	1.06	1.61	11
Vd_FPV_	Volume of distribution of favipiravir	L	10.4	10.5	8.12	14.5	18
CL_M1_	Clearance of favipiravir hydroxide	L/h	16.30 FIXED				
Vd_M1_	Volume of distribution of favipiravir hydroxide	L	6.44 FIXED				
T_abs_	Absorption half-life	h	1.15	1.24	0.36	2.43	42
F	Absolute bioavailability		1 FIXED				
TRF	Renal function effect coefficient for CL_M1_		0.0095 FIXED				
*Pharmacodynamics*							
UAbase	Baseline serum uric acid level	mg/dL	4.43	4.44	3.74	5.06	7
T1/2_UA_	Uric acid elimination half-life	h	17.6	17.4	9.4	25.6	25
Imax	The maximum extent of inhibition effect		1 FIXED				
C50_FPV_	Protein unbound concentration of favipiravir producing 50% of the maximum inhibition effect	mg/L	126 FIXED				
C50_M1_	Protein unbound concentration of favipiravir hydroxide producing 50% of the maximum inhibition effect	mg/L	52 FIXED				
FUB_FPV_	Unbound protein fraction of favipiravir		0.5 FIXED				
FUB_M1_	Unbound protein fraction of favipiravir hydroxide		0.3 FIXED				
GAMMA_FPV_	Hill coefficient of turnover Imax model on favipiravir		0.41	0.40	0.19	0.64	27
GAMMA_M1_	Hill coefficient of turnover Imax model on favipiravir hydroxide		1 FIXED				
Between-subject variability (BSV)							
CL_FPV_/F			0.483	0.465	0.295	0.586	16
Vd_FPV_			0 FIXED				
CL_M1_			0.212 FIXED				
Vd_M1_			0 FIXED				
T_abs_			0 FIXED				
F			0 FIXED				
UAbase			0.263	0.251	0.178	0.313	14
T1/2_UA_			0 FIXED				
Imax			0 FIXED				
C50_FPV_			0 FIXED				
C50_M1_			0 FIXED				
FUB_FPV_			0 FIXED				
FUB_M1_			0 FIXED				
GAMMA_FPV_			0.920	0.848	0.009	1.588	48
GAMMA_M1_			0 FIXED				
Residual unidentified variability (RUV)							
RUV_PROP_FPV_	Proportional residual unidentified variability of favipiravir concentration		0.445	0.427	0.267	0.604	22
RUV_ADD_FPV_	Additive residual unidentified variability of favipiravir concentration	mg/L	0.135	0.172	0.028	0.194	328
RUV_PROP_M1_	Proportional residual unidentified variability of favipiravir hydroxide concentration		0 FIXED				
RUV_ADD_M1_	Additive residual unidentified variability of favipiravir hydroxide concentration	mg/L	0.241 FIXED				
RUV_PROP_UA_	Proportional residual unidentified variability of uric acid		0 FIXED				
RUV_ADD_UA_	Additive residual unidentified variability of uric acid	mg/dL	1.300	1.270	1.010	1.470	10

CI, confidence interval; %RSE, relative standard error. BSV calculated from square root (sqrt) (NONMEM^®^ OMEGA). RUV estimated using THETA. 95% CI was estimated from 2.5 to 97.5 percentile of the bootstrap sample estimates.

## Data Availability

The data presented in this study are available from the corresponding author upon reasonable request. The underlying raw clinical data are stored in a secure database with restricted access and are not publicly available because public data sharing was not approved under the ethics approval and to protect patient privacy. Requests will be considered on a case-by-case basis, subject to institutional and ethical regulations.

## Supplementary Materials

The following supporting information can be downloaded at: https://www.mdpi.com/article/10.3390/ph19071008/s1, Figure S1: Goodness-of-fit plots based on the final population pharmacokinetic model; Figure S2: Structural pharmacokinetic model for favipiravir and favipiravir hydroxide and pharmacodynamic model for uric acid.

## Abbreviations

The following abbreviations are used in this manuscript:

AUC	Area under the curve
BSV	Between-subject variability
CI	Confidence interval
CLcr	Creatinine clearance
CLcr_STD_	Standard CLcr
CL_FPV_/F	Clearance of favipiravir
CL_M1_	Clearance of favipiravir hydroxide
COVID-19	Coronavirus disease 2019
C50_FPV_	Protein unbound concentration of favipiravir producing 50% of the maximum inhibition effect
C50_M1_	Protein unbound concentration of favipiravir hydroxide producing 50% of the maximum inhibition effect
df	Degrees of freedom
Edrug	Pharmacodynamic model for favipiravir
FOCE-I	First-order conditional estimation method with interaction
FPV	Favipiravir
F	Absolute bioavailability
F_SIZE_	Factor for size
GAMMA_FPV_	Hill coefficient of turnover Imax model on favipiravir
GAMMA_M1_	Hill coefficient of turnover Imax model on favipiravir hydroxide
Imax	The maximum extent of inhibition effect
ka	Absorption rate constant
kout	Uric acid elimination rate constant
LLOQ	Lower limit of quantification
M1	Favipiravir hydroxide
OFV	Objective function value
pcVPC	Prediction-corrected visual predictive check
PDI	Inhibition of the uric acid elimination process
PI	Percentile interval
PPP and D	Population pharmacokinetic parameters and data method
PWR	Allometric exponent
RF	Renal function
Rin	Zero-order production rate (uric acid)
RUV	Residual unidentified variability
SFTS	Severe fever with thrombocytopenia syndrome
T_abs_	Absorption half-life
TBW	Total body weight
TRF	Renal function effect coefficient
T1/2_UA_	uric acid elimination half-life
UA	Uric acid
UAbase	Baseline serum uric acid level
Vd_FPV_	Volume of distribution of favipiravir
Vd_M1_	Volume of distribution of favipiravir hydroxide
VPC	Visual predictive check
ΔOFV	Difference in OFV
%RSE	Relative standard error
