# Supplementary figures and images for: Population Pharmacokinetic Analysis and Modelling of Serum Uric Acid Dynamics in Patients Treated with Favipiravir
Source: Pharmaceuticals (Basel). 2026 Jun 29;19(7):1008. doi: 10.3390/ph19071008 (PMC13416278; doi:10.3390/ph19071008)

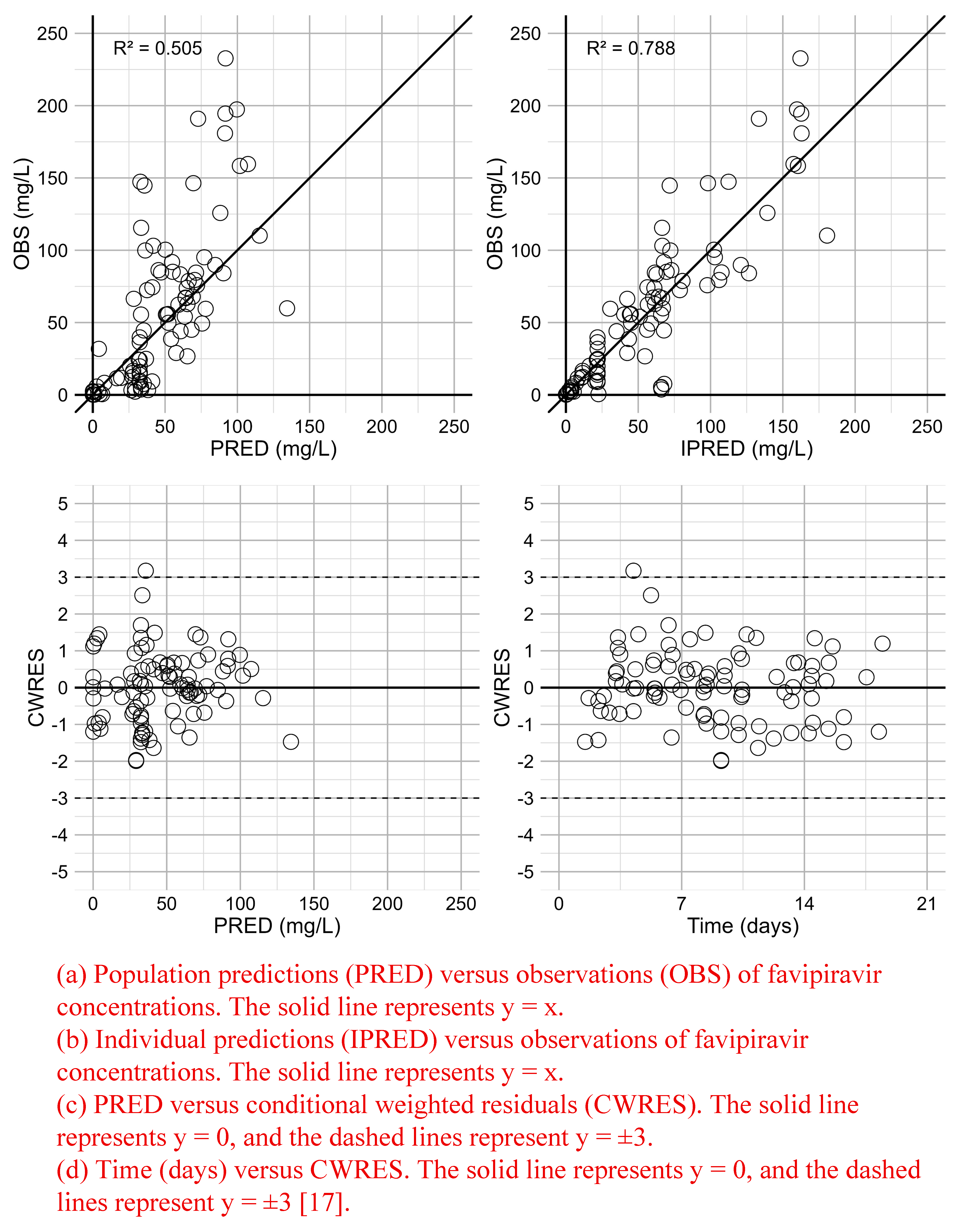

Supplement: Supplementary file 1 [file pharmaceuticals-19-01008-s001.zip › Figure S1.png]

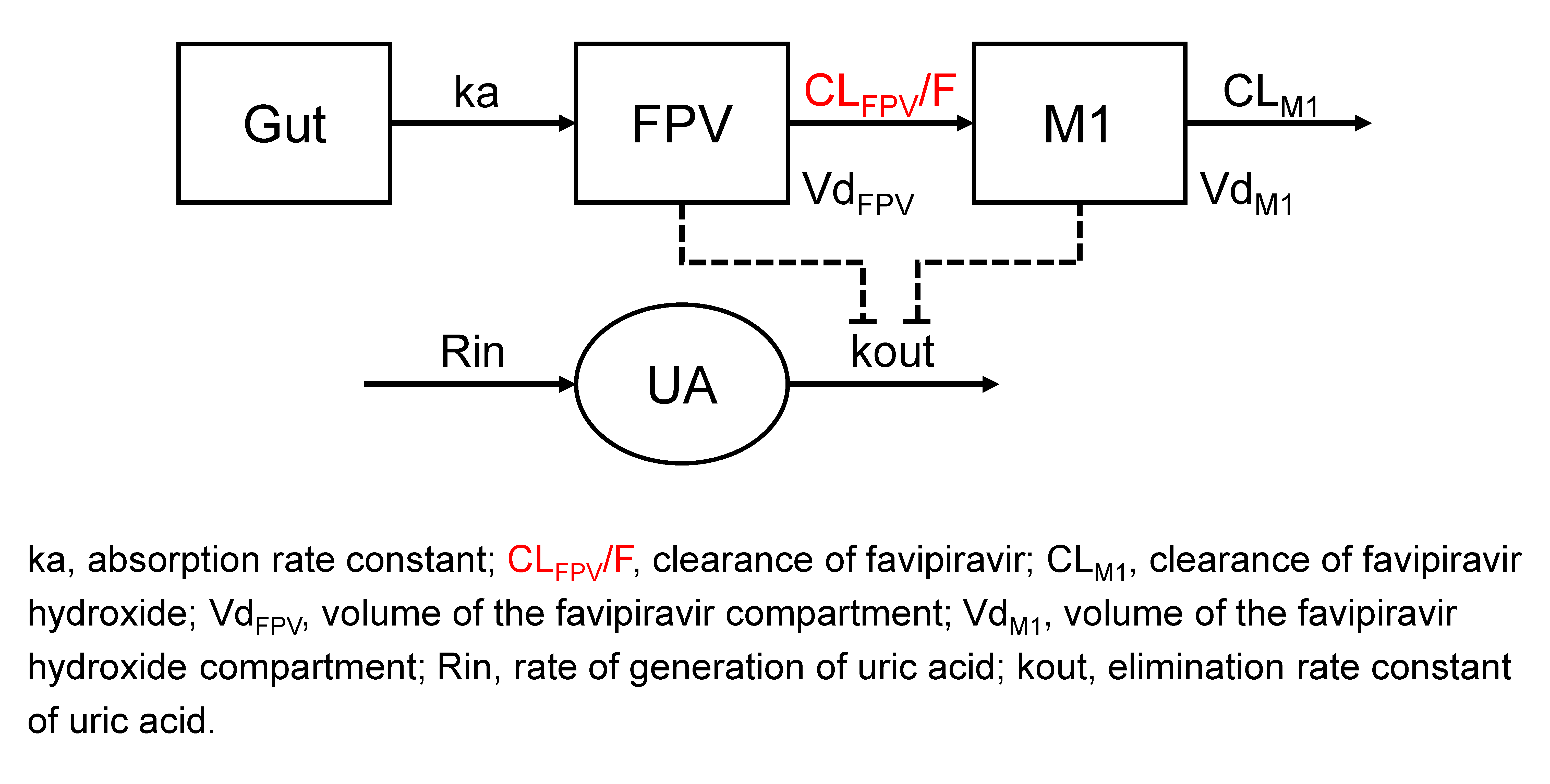

Supplement: Supplementary file 1 [file pharmaceuticals-19-01008-s001.zip › Figure S2.png]
